# Exploring Brain Derived Neurotrophic Factor and Cell Adhesion Molecules as Biomarkers for the Transdiagnostic Symptom Anhedonia in Alcohol Use Disorder and Comorbid Depression

**DOI:** 10.3389/fpsyt.2020.00296

**Published:** 2020-04-20

**Authors:** Lyudmila A. Levchuk, Elise M. G. Meeder, Olga V. Roschina, Anton J. M. Loonen, Anastasiia S. Boiko, Ekaterina V. Michalitskaya, Elena V. Epimakhova, Innokentiy S. Losenkov, German G. Simutkin, Nikolay A. Bokhan, Arnt F. A. Schellekens, Svetlana A. Ivanova

**Affiliations:** ^1^ Mental Health Research Institute, Tomsk National Research Medical Center of the Russian Academy of Sciences, Tomsk, Russia; ^2^ Department of Psychiatry, Donders Institute for Brain, Cognition, and Behavior, Radboudumc, Nijmegen, Netherlands; ^3^ Unit of PharmacoTherapy, Epidemiology, & Economics, Groningen Research Institute of Pharmacy, University of Groningen, Groningen, Netherlands; ^4^ Nijmegen Institute for Scientist-Practitioners in Addiction (NISPA), Radboud University Nijmegen, Nijmegen, Netherlands

**Keywords:** biomarker, transdiagnostic, brain-derived neurotrophic factor, cell adhesion molecule, depressive disorder, addiction, alcohol use disorder, anhedonia

## Abstract

**Background:**

Alcohol Use Disorder (AUD) and depressive disorder often co-exist and have a shared heritability. This study aimed to investigate Brain-Derived Neurotrophic Factor (BDNF) and three Cell Adhesion Molecules (CAMs) as transdiagnostic biomarkers in AUD and depression co-morbidity.

**Methods:**

In a cross-sectional study, patients with AUD (n=22), AUD and depression (n=19), and healthy controls (n=20) were examined. Depression and anxiety severity were assessed using the Hamilton Depression Rating Scale and the Hamilton Anxiety Rating Scale. Anhedonia, alcohol use and dependence, craving, and social adaptation were assessed through self-report questionnaires. BDNF and CAM concentrations in peripheral serum were measured after overnight fasting using a Luminex assay. After controlling for age and gender, biomarker levels were compared across groups. The association between biomarker concentrations and symptom severity scales were explored using correlation and multiple regression analyses.

**Results:**

BDNF and Neuronal CAM were lower in patients with AUD with and without depression compared to healthy controls. No differences were observed for Vascular CAM-1 and Interstitial CAM-1. BDNF correlated negatively with anhedonia levels. BDNF, age and gender together explained 21% of variability in anhedonia levels.

**Conclusion:**

This pilot study suggests that peripheral levels of BDNF and NCAM might be reduced in AUD with and without comorbid mood disorder. Since low BDNF levels were associated with self- reported anhedonia across these conditions, BDNF and anhedonia might reflect transdiagnostic aspects involved in AUD and depression.

## Introduction

Psychiatric disorders, including addictive and depressive disorders, are widespread in the world and pose a significant public health burden. Alcohol use disorder (AUD) and depressive disorder are among the most prevalent psychiatric conditions. The 12-month prevalence of alcohol use disorder in the Russian Federation is as high as 20.9%, compared to 13.9% in the USA. The prevalence rates of depressive disorders in these countries are 5.9% and 5.5%, respectively (1, 2). Moreover, AUD and depression often co-occur, with the presence of either disorder doubling the risks of the other (3). Importantly, this common co-morbidity aggravates clinical symptoms, worsens prognosis, and limits therapeutic response of both conditions (4).

Several authors suggest shared etiological mechanisms mediating frequent co-morbidity of psychiatric disorders, including AUD and depression. Indeed, genetic studies indicate significant shared heritability of AUD and depression (5). In line with the Research Domain Criteria (RDoC) several transdiagnostic symptom domains are shared in AUD and depression (6). These RDoC domains include symptoms of positive valence (e.g. reward processing and anhedonia), negative valence (e.g. anxiety), social functioning (e.g. social communication and perception), cognition, and arousal (7). In order to further our understanding of potential transdiagnostic mechanisms contributing to co-occurrence of AUD and depression, insight in shared biological abnormalities is indispensable.

Peripheral biomarkers might shed light on shared biological abnormalities in AUD and depression. For instance, brain-derived neurotrophic factor (BDNF) has been proposed to play a major role in the pathophysiology of depression, and has also been related to AUD (8, 9). According to the neurotrophin hypothesis, stress might decrease BDNF levels, which could lead to decreased neuronal plasticity, resulting in depression (10). Several meta-analyses have confirmed that serum BDNF concentrations are decreased in untreated depressed patients and normalized by antidepressant treatment (11–14). However, contradictory results regarding the relationship between BDNF, BDNF genotype polymorphisms and depression exist (15).

Though BDNF in AUD has received less attention, a meta-analysis also found lower serum BDNF levels in active alcohol users compared to controls (16). This difference was most pronounced after detoxification (16). Yet, genetic studies did not support the role of BDNF in AUD (17). It is unclear to what extent co-morbid conditions, such as depressive disorder affect the relationship between BDNF and AUD. Therefore, a more transdiagnostic approach is required, studying the role of BDNF in single and co-morbid cases of depressive disorder and AUD.

Several other peripheral biomarkers are of interest in the context of depressive disorder and AUD co-morbidity. For instance, genome wide association studies on substance use disorders (SUD) have implicated genetic variants in cell adhesion molecule (CAM) genes in SUD (18). Moreover, addiction has been linked to decreased polysialylated (PSA-)NCAM levels in multiple animal studies (19–22). Variation within CAM genotypes has also been associated with treatment response in patients with depressive disorders (23). Furthermore, post-mortem studies revealed increased levels of CAM gene expression in prefrontal cortex in patients with depressive disorder, and increased CAM levels have been observed in the cerebrospinal fluid of patients with depressive disorder (24–26). CAMs are among the most abundant proteins in the nervous system, and play essential roles in synaptic plasticity and functioning (27). Interestingly, diverse CAMs seem to interact with BDNF. For example, BDNF was shown to restore long-term potentiation in the hippocampus of polysialylated (PSA-) NCAM deficient mice (28). Depression is associated with both decreased levels of BDNF and PSA-NCAM levels (29). In contrast, vascular cell adhesion molecule-1 (VCAM-1) and intracellular adhesion molecule-1 (ICAM-1) might have opposing effects to BDNF. VCAM-1 was inversely correlated with serum BDNF in healthy men, and BDNF inhibited expression of ICAM-1 in interleukin-1b-treated human endothelial cells *in vitro* (30, 31). In patients suffering from depression, soluble forms of ICAM-1 and VCAM-1 were increased, as opposed to reduced levels of BDNF (32).

Little is known on levels of BDNF and CAMs in patients with co-occurrence of AUD and depressive disorder. Therefore, in this pilot study we measured levels of both BDNF and CAMs in patients with AUD with and without depressive disorder, and controls. We combined these data with measures of severity of both disorders. We hypothesized that 1) BDNF and NCAM levels were decreased, and that VCAM-1 and ICAM-1 levels were increased in patients with AUD (with and without co-morbid depression); and 2) that BDNF and NCAM levels were decreased, and VCAM-1 and ICAM-1 levels were increased in patients with AUD with depression co-morbidity as compared to AUD only. Furthermore, we expected biomarkers to correlate with symptom severity levels. Finally, we explored the relationship of BDNF and CAM biomarkers with transdiagnostic symptom domains anhedonia, anxiety, and social disfunction.

## Materials and Methods

### Design

In a cross-sectional transdiagnostic case-control study the relationship between phenotype measures of depression and AUD were compared across two diagnostic groups (AUD with and without depressive disorder), and healthy controls. The study was carried out in accordance with the Code of Ethics of the World Medical Association (Declaration of Helsinki 1975, revised in Fortaleza, Brazil, 2013), and approved by the Institutional Medical Review Board (Protocol of the local ethics committee at the Research Institute of Mental Health № 101 from 13 June 2017). All participants provided written informed consent.

### Participants

Participants with AUD with depressive disorder (n=19) and without depressive disorder (n=22), were recruited from the departments of affective and addictive disorders of Mental Health Research Institute of the Tomsk National Research Medical Center. Inclusion criteria were: a diagnosis of AUD (F10.2) with or without depressive episode/dysthymia (F31, F32, F33, F34.1), according to ICD-10; ages 18–60 years. We excluded patients with other comorbid mental disorders, for instance schizophrenia, intellectual disability, and alcoholic psychoses, and patients with acute physical diseases. The screening for relevant pathology for in/exclusion of subjects was performed through clinical assessment by three trained psychiatrists (OR, GS, and NB) on the first day of admission. Alcohol history and withdrawal severity were assessed correspondingly. Patients in the state of withdrawal received benzodiazepine therapy to alleviate withdrawal symptoms. The duration of alcohol withdrawal as estimated by the treating psychiatrist was on average 2–4 days after admission.

The control group consisted of 20 healthy volunteers recruited through local advertisements at the MHRI and Tomsk University. Healthy individuals were screened using a self-report questionnaire. The questionnaire screens for both physical and mental pathology, e.g. endocrine, neurological, gynecological and psychiatric disorders.

### Measurements

#### Phenotype Measures

Depressive symptoms were assessed using the 17 item Hamilton Depression Rating Scale (HAMD-17), the most widely used clinician-rated measure of depression severity, with scores ranging from 0 to 52 (33). Higher scores indicate higher severity levels of depression. The HAMD-17 is part of an official Russian translation of the SIGH-SAD by K.V. Danilenko and N.K. Danilenko (34). The HAMD is a reliable tool for the assessment of depression severity, applied across the globe, though psychometric properties of the official Russian version have not been published (35). Anxiety symptoms were measured using the Hamilton Anxiety Rating Scale (HAM-A), a semi-structured interview. The HAM-A has a score range from 0 to 56 (36). Higher scores indicate higher severity levels of anxiety. The remaining transdiagnostic symptom domains were assessed using Russian translations of commonly applied self-report questionnaires. Anhedonia, a core level of depression, was assessed using the Snaith–Hamilton Pleasure Scale (SHAPS) (37). This is a 14-item questionnaire, with a total score range from 0 to 14. Alcohol use and dependency were measured using the Alcohol Use Disorders Identification Test–Consumption (AUDIT-C) (38). The total score on this 10 item questionnaire ranges from 0 to 40. Craving was assessed using the Obsessive Compulsive Drinking Scale (OCDS), which consist of 7 items related to drinking compulsions (behavior) and 7 items related to drinking obsessions (thoughts), total score 0 to 40 (39). The Social Adaptation Self-evaluation Scale (SASS) was used to measure the level of social adaptation (40). The SASS consists of 21 items, and has a total score ranging from 0 to 60. For all questionnaires except the SASS, higher scores indicate higher symptom severity. For the SASS, higher scores signify better social adjustment, i.e. less severe psychopathology. All self-report questionnaires were common Russian translation of the originals (verified by back-translation into English). The psychometric tests were performed during the first week of admission, after remission of withdrawal or intoxication symptoms.

#### Biomarkers

Peripheral venous blood was collected from each subject at 8.00–9.00 a.m. on the morning after hospital admission, after eight hours of overnight fasting before intake of any food or medication. Blood was sampled in BD Vacutainer tubes with coagulation activator and centrifuged at 2000 rcf at 4 ° C for 20 min. Serum samples were stored at -80^o^C until they could be analyzed.

Concentrations of analytes were determined on the MAGPIX multiplex analyzer (Luminex, USA) using xMAP® Technology. Panel HNDG3MAG-36K by MILLIPLEX® MAP (Merck, Darmstadt, Germany) was used to determine the levels of the markers BDNF, sICAM-1, sVCAM-1, and NCAM. Further details are described in the **Supplementary Material**. The detected information is processed by special Luminex xPONENT® software, with subsequent export of data to the MILLIPLEX® Analyst 5.1 program.

### Data Analysis

Mean serum biomarker levels were compared among the groups using analysis of covariance (ANCOVA), controlling for the confounding variables age and gender. Planned contrast analysis was used to make individual comparisons between groups. Partial correlations were conducted between serum biomarker levels and the symptom severity scales, controlling for age and gender. Biomarkers and symptom severity measures which displayed significant correlations were selected to be further examined by regression analysis. To investigate the extent to which these biomarkers and the variables age and gender contribute to symptom severity, multiple regression analyses was performed. All analyses were carried out using SPSS version 25 Windows. The significance level for all analyses was set at 2-sided P<0.05.

## Results

The demographic and clinical characteristic across the different patient groups are shown in **Table 1**. The control group was younger (37.2 versus 45.6 years, p<.001) and more often female (75.0% versus 14.6%, p<.001) as compared to the patient groups. In the group patients with AUD and a comorbid mood disorder, four had a unipolar depressive disorder (21.1%), four a recurrent depressive disorder (21.1%), eight dysthymia (42.1%), and three a bipolar depressive disorder (15.8%). Though patients with AUD and comorbid mood disorder displayed higher levels of depression and anxiety severity as compared to the patients with only AUD (mean HAMD-17 18.2 ± 8.4 versus 7.6 ± 5.0 respectively, p<.001; mean HAMA 18.2 ± 9.9 versus 8.1 ± 5.9 respectively, p<.001), they showed similar severity levels across other symptom domains (e.g., mean SHAPS scores 1.7 ± 1.7 and 2.0 ± 2.0 respectively; p = .60).

**Table 1:** Characteristics of the participants.

	AUD(n = 22)	AUD+MD(n = 19)	p-value
Age (years, mean ± SD)	48.1 ± 9.7	42.7 ± 8.8	.22
Sex (male, n, %)	20 (90,9)	15 (78,9)	.28
Duration of illness (years, mean ± SD)			
AUD	13.4 ± 6.2	14.3 ± 7.6	.66
MD	–	7.9 ± 7.3	–
Current episode (months, mean ± SD)			
AUD	12.3 ± 12.8	16.0 ± 27.1	.57
MD	–	24.4 ± 36.9	–
Symptom severity scores (mean ± SD)		
HAMD-17 SHAPS	1.7 ± 1.7 37.6 ± 7.2	18.2 ± 8.4 2.0 ± 2.0	.000*** .60
SASS^†^	7.6 ± 5.0	37.9 ± 6.4	.93
AUDIT-C	23.3 ± 6.9	26.0 ± 6.4	.21
OCDS	24.6 ± 10.0	33.4 ± 11.8	.01*
HAMA	8.1 ± 5.9	18.2 ± 9.9	.000***

AUD, alcohol use disorder; MD, mood disorder; p, 2-tailed p-value for ANOVA or chi square test; SD, standard deviation; HAMD-17, Hamilton Depression Rating Scale; SHAPS, Snaith–Hamilton Pleasure Scale; SASS, Social Adaptation Self-evaluation Scale; AUDIT-C, Alcohol Use Disorders Identification Test; OCDS, Obsessive Compulsive Drinking Scale; HAMA, Hamilton Anxiety Rating Scale; *p <.05, **p <.01, ***p<.001, ^†^higher scores signify less symptom severity.

The mean serum BDNF level was lower in patients with AUD with and without comorbidity in comparison to healthy controls (mean concentration 2.685 ± .29 and 2.692 ± .29 versus 4.069 ± .33 ng/ml respectively, p = .009). There were no differences between the two patient samples. Mean serum NCAM levels were lower in patients with AUD with and without comorbid mood disorder compared to healthy controls (mean concentration 36.062 ± 2.83 and 28.585 ± 2.37 versus 38.198 ± 2.62 ng/ml respectively, p = .027). This effect was mainly driven by lower levels of NCAM in patients with AUD only, as compared to patients with AUD and a mood disorder. The adjusted mean levels of sICAM1 and sVCAM1 did not significantly differ between the diagnostic groups. Mean biomarker levels across the different study groups, controlling for age and gender are reported in **Table 2**.

**Table 2:** Adjusted mean peripheral biomarker levels across groups, controlling for age and gender.

	Controls	AUD	AUD+MD	df_M_, df_R_	F-value	p-value omnibus test	p-value planned contrast	
							I	II
BDNF [ng/ml, mean (SE)]	4.069 (.33)	2.692 (.29)	2.685 (.29)	2, 56	5.17	.009**	.002**	.99
NCAM [ng/ml, mean (SE)]	38.198 (2.62)	28.585 (2.37)	36.062 (2.83)	2, 55	3.85	.027*	.09	.022*
sICAM1 [ng/ml, mean (SE)]	15.272 (1.42)	15.141 (1.39)	17.153 (1.26)	2, 52	.80	.46	–	–
sVCAM1 [ng/ml, mean (SE)]	117.816 (8.48)	121.540 (7.47)	122.542 (7.37)	2, 56	.08	.92	–	–

AUD, alcohol use disorder; MD, mood disorder; dfM, model degrees of freedom; dfR, residuals degrees of freedom; F, F-ratio; I, controls vs all patients (both AUD and AUD+MD); II, patients with AUD versus patients with AUD+MD; BDNF, brain derived neurotrophic factor; NCAM, neural cell adhesion molecule; sICAM1, intracellular adhesion molecule-1; sVCAM1, vascular cell adhesion molecule-1; SE, standard error; *p <.05, **p <.01.

A significant negative correlation (controlling for age and gender) was found between BDNF and SHAPS scores, indicating the lower the BDNF level, the more severe the anhedonia (see **Supplementary** **Table 1**). There were no other significant correlations observed.

Multiple regression analysis confirmed the association between BDNF and anhedonia, indicating BDNF, age and gender together explained 21% of variability in anhedonia levels (see **Table 3**). The β-value coefficient indicated that a unit fall in BDNF level equals an average rise of 0.44 units in SHAPS score (p = .008). Furthermore, also female gender was significantly associated with higher anhedonia levels (β = .12, p = .026).

**Table 3:** Results of multiple regression analysis to assess the nature of the independent relationship between BDNF level and anhedonia severity (Snaith–Hamilton Pleasure Scale).

Model	Predictor variable	B ± SE	β	p-value	Model R^2^
1	Age	0.02 ± 0.03	.09	.59	.04
	Gender (female)	1.62 ± 0.94	.29	.09	
2	Age	0.02 ± 0.03	.36	.450	.21*
	Gender (female)	2.01 ± 0.86	.12	.026*	
	BDNF	-1.12 ± 0.40	-.44	.008**	

B, unstandardized coefficient; SE, standard error; β, standardized coefficient; R^2^, proportion of predictable variance; BDNF, brain derived neurotrophic facto; *p <.05, **p <.01.

## Discussion

This pilot study investigated BDNF and CAM levels transdiagnostically in patients with AUD with, and without depression, and healthy controls. The results suggest lower BDNF and NCAM levels in patients with AUD with and without comorbid depression, compared to healthy controls. Lower BDNF levels were associated with higher levels of anhedonia. There were no other relationships observed between biomarker and symptom levels.

Our findings on reduced BDNF levels in AUD and mood disorders are in line with our hypothesis and several human studies indicating that both disorders are associated with decreased levels of BDNF (11–13, 16). However, contradictory findings have been observed regarding serum BDNF and depression, and the evidence for reduced BDNF in patients with AUD remains limited (41, 42).

We observed a negative correlation of BDNF levels with anhedonia, but not with the severity of depressive symptoms in general. This might be explained by the high levels of dysthymia patients in the comorbid group. It has previously been shown that BDNF levels are indeed decreased in patients with major depressive disorder, but not in patients with dysthymia. This might have contributed to a lack of association between BDNF levels and general depression symptom severity levels (43). Furthermore, it has been suggested that dopaminergic depletion, as commonly observed in addicted patients, is associated with anhedonia (44). Depressive co-morbidity in patients with AUD may thus be characterized by more pronounced levels of anhedonia, as compared to other symptom domains of depression (e.g. anxiety or negative mood). The association between BDNF and anhedonia levels in absence of an association between BDNF and general depression severity could indicate that in our comorbid sample anhedonia might indeed have been the most affected symptom domain of depression.

Though our findings should be considered preliminary due to limited sample size and the cross-sectional nature of our pilot study impedes any causal inferences, it is tempting to speculate about potential mechanisms involved in our findings. Several animal studies show that BDNF deficiency reduces sucrose preference, a loss of sensitivity to reward which has been suggested to model anhedonia (45–48). Anhedonia is a core symptom of depression, and also plays an essential role in addictive disorders (49, 50). In our sample, peripheral BDNF levels were indeed specifically related with self-reported anhedonia levels in the combined sample. This suggests a potential transdiagnostic mechanism of low BDNF-related anhedonia in AUD depression co-morbidity.

Low levels of BDNF might be associated with the transdiagnostic symptom of anhedonia through alterations in ventral extrapyramidal circuits regulating motivation to reward-seeking (success leads to pleasure, hedonia) and distress-avoiding (success leads to happiness, euphoria) activities in both types of disorders (51). The activity of these circuits is regulated by ascending monoaminergic neurons originating within the midbrain, which are in turn controlled by an evolutionary well conserved system of the habenuloid complex (51). Glutamatergic lateral habenula-projecting globus pallidus neurons are involved in the evaluation of the results of reward-seeking activities and inhibit the motivation to continue them via ascending dopaminergic pathways to the ventral striatum (51). Previous findings using deep brain stimulation of the lateral habenula suggest that these glutamatergic neurons are sensitive to BDNF-induced neuroplastic changes (52).

The decreased serum NCAM levels in patients with AUD with and without comorbid depression, is in contrast with a clinical study which found increased PSA-NCAM1 expression in lethally intoxicated patients with opioid use disorder (53). Similar results were obtained by Cirielli and colleagues (54). However, it has been demonstrated in mice that decreased expression of PSA-NCAM is related to individual risk for alcohol-related behaviors (55). Moreover, several other animal studies suggest that both substance use disorder and depression are associated with lower NCAM levels (19–22, 29). This might suggest differential mechanisms involved in addiction to different substances. Furthermore, it has to be acknowledged that our study measured NCAM levels shortly after detoxification, while in the study of Weber et al. and Cirielli et al. patients were lethally intoxicated with illicit drugs (53, 54). It is unknown to what extent intoxication, withdrawal and abstinence affect peripheral biomarker levels, including NCAM (53, 56).

In contrast to our hypothesis, we did not find any differences in sICAM-1 and sVCAM-1 levels across the different groups. Since one clinical study did find elevated levels of both s-ICAM-1 and sVCAM-1 in 33 elderly patients with depression, this might be related to insufficient power in our study or less contribution of ischemia-induced inflammation in our patients with AUD and depression (32). Insufficient power could also explain the absence of any differences in BNDF and NCAM levels in patients with AUD and comorbid depression, as compared to AUD only.

The current pilot study should be considered in the light of several limitations. First, our pilot study included only about 20 individuals per group, and the control group was younger and had more females in comparison with the two patient groups. Despite controlling for age and gender statistically, any confounding effects of age and gender cannot be fully ruled out. The current findings thus await confirmation in substantially large and well-matched samples. Secondly, our design did not include a group of patients with mood disorders only. It would be of great interest to investigate BDNF, CAM, and anhedonia levels in a sample of patients with only mood disorders in further research. Moreover, we cannot be certain that the reliability and validity of the Russian translated questionnaires is comparable with their characteristics within USA citizens. Future studies should test the psychometric characteristics of these translated instruments in Russian populations in comparison to those of other nations. Another limitation of the current study is its cross-sectional nature, impeding causal inferences about the relationship between biomarkers and psychopathology. Future studies using multi-level biomarkers, including genomic, transcriptomic and metabolomic markers might shed further light on the observed relationships. More experimental designs, including animal studies, and application of neuro-imaging techniques, are needed to gain better insight in causal mechanisms involved. Furthermore, the relationship between peripheral biomarker levels and brain tissue levels has been suggested in an animal study, but awaits confirmation in future studies (57).

In conclusion, this pilot study suggests that peripheral levels of BDNF and NCAM might be reduced in AUD with and without comorbid mood disorder. Since low BDNF levels were associated with self-reported anhedonia across conditions, future studies might further explore anhedonia as a transdiagnostic symptom in AUD and depression. Furthermore, future studies should further investigate potential mechanisms involved in the association between BDNF, NCAM, and AUD and depression.

## Data Availability Statement

The datasets generated for this study are available on every reasonable request to SI (ivanovaniipz@gmail.com), following approval of the Board of Directors of the MHRI, in line with local guidelines and regulations.

## Ethics Statement

The studies involving human participants were reviewed and approved by the Institutional Medical Review Board (Protocol of the local ethics committee at the Research Institute of Mental Health № 101 from 13 June 2017). The patients/participants provided their written informed consent to participate in this study.

## Author Contributions 

AL, AS, and SI instigated and designed the study. SI coordinated and supervised the study. EMM designed and performed the statistical analysis. LL, OR, GS, and SI wrote the study protocol. LL and OR monitored the study. OR, IL and GS collected clinical data. EVM and EE collected blood samples. AB measured the biomarkers in serum. LL and OR recorded all data in an Excel database. NB supervised the clinical work. SI supervised the technical work. LL, OR, EMM, AL, AS, and SI wrote the manuscript. EMM and AL reviewed and adapted the manuscript. All authors read the paper and agree with its content.

## Funding

The study was funded by a grant from the Russian Science Foundation (project No. 19-15-00023). The number of affiliations of the Russian contributors is required to be limited to a single (main) institution. This grant is applied to pay for reagents and salaries of Russian (co-)authors, as well as the publication fee.

## Conflict of Interest

The authors declare that the research was conducted in the absence of any commercial or financial relationships that could be construed as a potential conflict of interest.
