# Supplementary material for: Exploring Brain Derived Neurotrophic Factor and Cell Adhesion Molecules as Biomarkers for the Transdiagnostic Symptom Anhedonia in Alcohol Use Disorder and Comorbid Depression
Source: Front Psychiatry. 2020 Apr 20;11:296. doi: 10.3389/fpsyt.2020.00296 (PMC7184244; doi:10.3389/fpsyt.2020.00296)
Supplement: Supplementary file 1 [file DataSheet_1.pdf]

Supplementary methods

Immunoassay technique

Serum biomarker concentrations were measured using The MILLIPLEX® MAP Human Neurodegenerative Magnetic Bead Panel 3 (HNDG3MAG-36K), a kit of 10 analytes including BDNF, NCAM, sICAM-1 and sVCAM-1.(1) This panel is based on the Luminex® xMAP® technology, a multiplex assay that uses microspheres to simultaneously measure multiple proteins in a single experiment.(2) Capture antibodies bind the analytes and couple them to the microspheres, which are coated with precise concentrations of fluorescent dyes.(2) Detection antibodies then also bind to the analytes, and a fluorescently labeled reporter molecules binds to the detection antibodies – a method known as sandwich immunoassay.

First, serum samples were diluted 100-fold using an assay buffer provided by the manufacturer.(1) Second, wells were washed three times with 200 µL assay buffer. Third, 25 µL standard, control and sample dilutions were loaded on the appropriate wells. Also, 25 µL assay buffer was added to background and sample wells, 25 µL matrix solution was added to background, standard and control wells and 25 µL of antibody-immobilized microspheres was added to all wells. Next, the panels were incubated on a plate shaker at 4°C for at least 16 hours.(1) After that, the contents were removed and all plates were rinsed three times with 200 µL wash buffer. Twenty-five microliters of biotinylated detection antibody were added and the plates were incubated for an hour at room temperature. Thereafter, 25 µL of streptavidin-phycoerythrin - a fluorescently labeled molecule which binds to biotin – was added and the plates were incubated for another 30 minutes. Finally, contents were removed again, wells were washed three times with 200 µL wash buffer and beads were resuspended in 100 µL drive fluid on a plate shaker for 5 minutes.(1)

Plates were read on MAGPIX® with xPONENT® software.(2) Median Fluorescent Intensity (MFI) data were saved and analyzed with a 5-parameter logistic curve-fitting method for calculating analyte concentrations in samples.

References

1. Millipore Corporation. Human Neurodegenerative Disease Magnetic Bead Panel 3 96-Well Plate Assay. Cat. # HNDG3MAG-36K. Billerica, MA 01821 USA. 2013. http://wwwmerckmilliporecom/NL/en/product/MILLIPLEX-MAP-Human-Neurodegenerative-Disease-Magnetic-Bead-Panel-3-Neuroscience-Multiplex-Assay,MM_NF-HNDG3MAG-36K.

2. https://www.luminexcorp.com/xmap-technology/.

Supplementary tables

Supplementary Table 1. Partial correlation coefficients, between biomarkers and symptom severity levels controlling for age and gender.

	**HAMD-17**		**SHAPS**		**AUDIT-C**		**OCDS**		**SASS**		**HAMA**	
	r	p	r	p	r	p	r	p	r	p	r	P
**BDNF**	-.06	.74	**-.45**	**.008****	-.03	.86	-.05	.75	-.06	.75	.09	.61
**NCAM**	.09	.59	.25	.17	-.05	.78	-.09	.58	-.06	.73	-.12	.49
**sICAM1**	.12	.51	-.14	.48	-.02	.91	-.09	.59	.03	.89	.08	.63
**sVCAM1**	-.02	.92	.02	.93	-.03	.85	-.08	.63	.28	.12	-.14	.39

HAMD-17 = Hamilton Depression Rating Scale, SHAPS = Snaith–Hamilton Pleasure Scale, AUDIT-C = Alcohol Use Disorders Identification Test, OCDS = Obsessive Compulsive Drinking Scale, SASS = Social Adaptation Self-evaluation Scale, HAMA = Hamilton Anxiety Rating Scale, r = Spearman's correlation coefficient, p = 2-tailed significance, * p <.05, ** p <.01, BDNF = brain derived neurotrophic factor, NCAM = neural cell adhesion molecule, sICAM1 = intracellular adhesion molecule-1, sVCAM1 = vascular cell adhesion molecule-1.
